# A Soft Robot Tactile Finger Using Oxidation-Reduction Graphene–Polyurethane Conductive Sponge

**DOI:** 10.3390/mi15050628

**Published:** 2024-05-07

**Authors:** Hangze Li, Chaolin Ma, Jinmiao Chen, Haojie Wang, Xiao Chen, Zhijing Li, Youzhi Zhang

**Affiliations:** 1 School of Mechanical and Electrical Engineering, Wenzhou University, Wenzhou 325025, China; lihangze2023@163.com (H.L.); mchaolin2023@163.com (C.M.); chenjinmiao22@163.com (J.C.); wanghaojie020@163.com (H.W.); lscx09@163.com (X.C.); 2 School of Information and Electrical Engineering, Hunan University of Science and Technology, Xiangtan 411201, China; lizhijingwei@163.com

**Keywords:** soft robot tactile finger, highly sensitivity, adjustable range, robotic grasping

## Abstract

Currently, intelligent robotics is supplanting traditional industrial applications. It extends to business, service and care industries, and other fields. Stable robot grasping is a necessary prerequisite for all kinds of complex application scenarios. Herein, we propose a method for preparing an elastic porous material with adjustable conductivity, hardness, and elastic modulus. Based on this, we design a soft robot tactile fingertip that is gentle, highly sensitive, and has an adjustable range. It has excellent sensitivity (~1.089 kpa^−1^), fast response time (~35 ms), and measures minimum pressures up to 0.02 N and stability over 500 cycles. The baseline capacitance of a sensor of the same size can be increased by a factor of 5–6, and graphene adheres better to polyurethane sponge and has good shock absorption. In addition, we demonstrated the application of the tactile fingertip to a two-finger manipulator to achieve stable grasping. In this paper, we demonstrate the great potential of the soft robot tactile finger in the field of adaptive grasping for intelligent robots.

## 1. Introduction

With the continuous development of artificial intelligence [1] and Internet of Things [2] in the past few years, flexible tactile sensors [3,4,5,6,7,8] have become one of the hot spots of research in the field of robotics, attracting many scholars from different fields. At the present time, mobile robots have become an essential part of the development of automation in various industries. Robotic grasping is the key to realizing tasks such as robotic sorting and assembly and is an important capability of collaborative robots. Recently, it has become the focus of researchers.

So far, researchers have developed many types of tactile sensors based on various conductive principles, such as piezoelectric [9,10,11], piezoresistive [12,13], photovoltaic [14,15], and capacitive principles [16,17,18,19], etc. Capacitive sensors are among the most widely researched types of tactile sensors and are characterized by high sensitivity, fast response, and low delay. They represent a hot spot for sensor research. Nowadays, researchers have used some new materials and processes. For example, Miao et al. [20] developed a flexible capacitive sensor based on a PDMS substrate, and a 3D conductive nanofiber porous sponge consisting of electrostatically spun PVDF [21,22,23] short nanofibers and rGO sheets was prepared via freeze-drying treatment of PDMS, which improves the durability of the sensor. PDMS [24] can enhance the elasticity and durability of sponges. This sensor can characterize large-scale and microscale piezoresistive properties. Amir et al. [25] proposed a low-cost multi-touch sensor based on capacitance change. This new sensor is very flexible and easy to manufacture, making it a suitable choice for soft robotics applications. Jun [26] et al. developed a novel sensor to be used in a robotic fingertip, reducing the complexity of control and greatly enhancing the way the gripper interacts with the object.

For flexible capacitive pressure sensors, much of the sensor’s sensitivity depends on the material. So far, researchers have found numerous materials to be used as electrodes for sensors; for example, Wang [27] et al. prepared a flexible electrically conductive polymer sponge composite with a lotus-leaf-inspired microstructure prepared by anchoring carbon nanotubes onto the skeleton of the polymer sponge with the assistance of ultrasonication and simultaneous non-solvent-induced phase separation (NIPS); and Cho [28] et al. fabricated a flexible tactile force sensor using conductive ink and silicone elastomers to use it in a real-time robotic feedback control system. In addition, materials chosen for sensor electrodes include conductive particles [29], organic field-effect transistors [30,31], liquid metals [32,33], hydrogels [34], etc. These sensors have achieved significant results in a range of cutting-edge devices such as smart manipulators [35], medical monitoring [36,37], and wearable electronics [38,39].

At present, most of the existing methods for the preparation of electrodes are not able to ensure extremely high sensitivity and response time of the haptic fingertip while adjusting the range and conductivity. Developing a simple but versatile technique for large-scale fabrication of flexible capacitive sensors with high elasticity, high sensitivity, and adjustable range remains challenging. In this paper, we demonstrate a practical and effective strategy by proposing a novel capacitive tactile sensor centered on flexible porous electrodes [40]. The elastic porous electrode uses a redox method to attach graphene [41,42,43,44,45] to a polyurethane sponge, and by adjusting the number of soaks and the firmness of the sponge, this, in turn, affects the range and sensitivity of the tactile fingertips. The material is structurally stable with excellent elastic and electrothermal properties and can be applied to flexible capacitive sensors with very high sensitivity (~1.089 kpa^−1^) and excellent response time (~35 ms) at low pressure. It has a broad potential in the field of robotic tactile fingertips and electronic skin.

## 2. Materials and Methods

### 2.1. Structure and Principle

**Figure 1:**
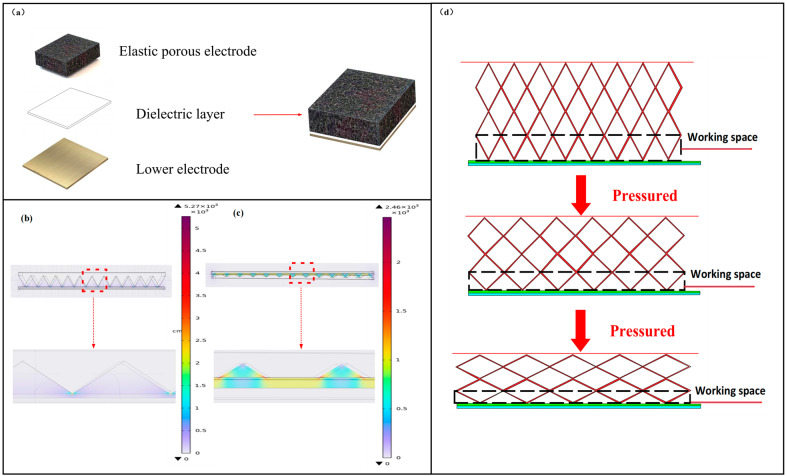
(**a**) Model diagram of tactile fingertip structure; (**b**) tactile fingertip electric field distribution based on elastic porous electrodes; (**c**) tactile fingertip electric field distribution based on elastic porous electrodes after full compression; (**d**) simplified model of a flexible haptic fingertip.

The new capacitive haptic fingertip consists of three parts: elastic porous electrodes, a dielectric layer, and lower electrode plate, and its simplified structure is shown in Figure 1a. When pressure is applied to the tactile fingertip, the distance between the upper and lower polar plates of the tactile fingertip decreases, increasing the strength of the electric field, which causes the capacitance value to rise. We can determine the applied pressure based on detecting the change in the capacitance value of the tactile fingertip.

To further investigate the working principle of capacitive haptic fingertips based on elastic porous electrodes, we performed simulations using COMSOL6.0 software. Assuming that the boundary of the air region is 1 m × 1 m × 1 m, the air region can be regarded as a completely insulated surface. We set the size of the elastic porous electrode before being uncompressed to 100 mm × 100 mm × 10 mm, the size after being compressed to 100 mm × 100 mm × 5 mm, and the size of the dielectric layer and lower electrode to 100 mm × 100 mm × 1 mm. We set the boundary potential of the elastic porous electrodes to be set to 1 V, and all the boundaries of the lower electrodes to be set to ground, while the boundaries of the air region are set to zero charge to simulate the electrostatic field. The electric field distribution of the uncompressed 3D porous material is shown in Figure 1b, from which it can be seen that the vast majority of the electric field lines are distributed in the region where the elastic porous electrodes are in contact with the dielectric layer, except for a small amount distributed in the boundary region on both sides, at which time the capacitance value of the haptic fingertip is 53.3 pf. The electric field distribution of the fully compressed 3D porous material is shown in Figure 1c, at which the polyurethane sponge produces an obvious elastic deformation, and the capacitance value increases, which can reach 167.5 pf, reaching three times of the initial capacitance value, but the densest region of the electric field is still the region where the elastic porous electrode is in contact with the dielectric layer. Based on the simulation results, we can conclude that most of the charge distribution in the upper electrode is on the lower surface of the elastic porous electrode, and the closer it is to the dielectric layer, the denser the charge distribution is. The contact area between the lower surface of the elastic porous electrode and the dielectric layer plays a dominant role in the variation of the capacitance value.

Because the contact area between the elastic porous electrode and the dielectric layer plays a dominant role, we define the lower surface area of the elastic porous electrode as the workspace, and its pressure detection principle is shown in Figure 1d. When the tactile fingertip is subjected to an external force, the elastic porous electrode is deformed by compression, and the contact area of the working space increases, leading to an increase in the capacitance value. When the external force is released, the contact area of the working space of the elastic porous electrode decreases, resulting in a decrease in the contact area between the elastic porous electrode and the dielectric layer, which causes a decrease in the capacitance value of the tactile fingertip. Thus, we can obtain the magnitude of the applied force by detecting changes in the capacitance of the tactile fingertip.

### 2.2. Fabrication of Qxidation-Reduction Graphene–Polyurethane Conductive Sponge

When pressure is applied to a capacitive haptic fingertip, we intend the haptic fingertip to be not only highly sensitive but also cushioned. This cushioning reduces the force impact and is important for improving the robustness of the sensors and protecting the robot. Therefore, a new elastic porous electrode was fabricated. The structure can either be used as an energy-absorbing element, which has a cushioning effect when detecting pressure and can reduce the effect of pressure, or as a sensitive element to produce different capacitance changes when the tactile fingertip is subjected to different pressures.

The materials used to prepare the oxidation-reduction graphene–polyurethane conductive sponge (hereafter replaced by RGO-PUF) included a honeycomb polyurethane sponge (purchased from Guangdong Dongguan Hengying Sponge Factory, Dongguan, China), graphene oxide solution at a concentration of 2 mg/mL (purchased from Sigma-Aldrich, Burlington, VT, USA), and hydriodic acid solution at a concentration of 55%–58% (purchased from Meryer Chemical Technology, Shanghai, China). All chemicals were used as received without further purification, and deionized water was used for all experiments and tests.

**Figure 2:**
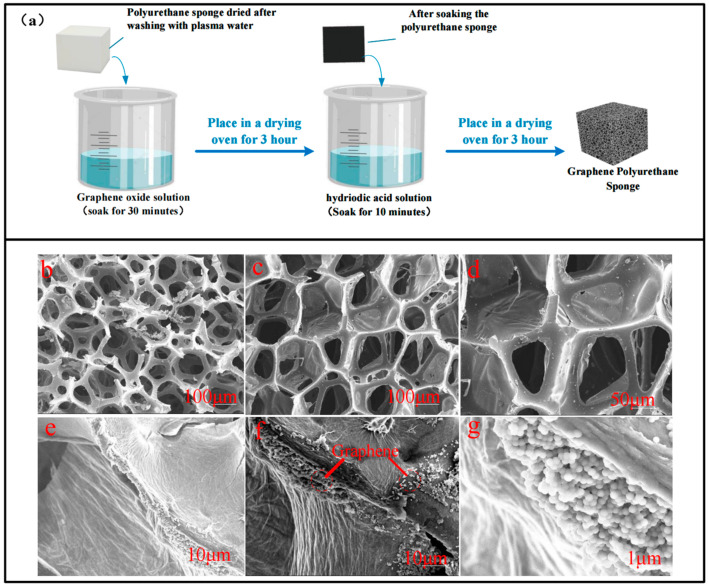
(**a**) Schematic diagram of RGO-PUF manufacturing process; (**b**) SEM image of pre-cleaned polyurethane sponge; (**c**) SEM images of RGO-PU; (**d**) enlarged morphology of RGO-PUF; (**e**) morphology of RGO-PUF skeleton soaked once; (**f**) morphology of RGO-PUF skeleton after five of soaks; (**g**) enlarged morphology of RGO-PUF skeleton.

First, we cut the cellular polyurethane sponge into a size of 20 mm × 20 mm × 5 mm. We cleaned it with plasma water and dried it in a drying oven at 60 °C for 2 h. The polyurethane sponge was then soaked in the graphene oxide solution for 30 min. The sponge should be squeezed several times during this period to allow for full absorption. During soaks, the mixed solution should be kept in a sealed beaker to minimize the effect of the volatilization of the dispersant on the concentration of the solution. The polyurethane sponge mixed with graphene oxide was then placed in a sealed beaker using a small amount of hydriodic acid, which was slowly dripped in with a dropper until it was completely submerged; then, it was gently pressed several times until it was completely absorbed. After soaking for a period of time, we rinsed the polyurethane sponge repeatedly until there was no color change when soaking the polyurethane sponge in water. Finally, it was dried in a drying oven at 60 °C for 3 h to obtain RGO-PUF. The RGO-PUF fabrication process is shown in Figure 2a.

The diameter of the honeycomb polyurethane sponge is around 50–200 μm, and the width of the skeleton is around 10–50 μm. Figure 2c–g shows the RGO-PUF made after the reaction of the redox method. The polyurethane sponge retains its 3D porous structure after soaking. Figure 2d shows an enlarged form of an RGO-PUF skeleton, where a number of granular substances can be seen on the skeleton of an RGO-PUF. We performed elemental analysis of RGO-PUFs using scanning electron microscopy (see Figure S2, Supporting Information). This shows that graphene particles have been wrapped around the surface of the 3D skeleton. From Figure 2e–g, we can see the graphene attached to the RGO-PUF skeleton more clearly.

**Figure 3:**
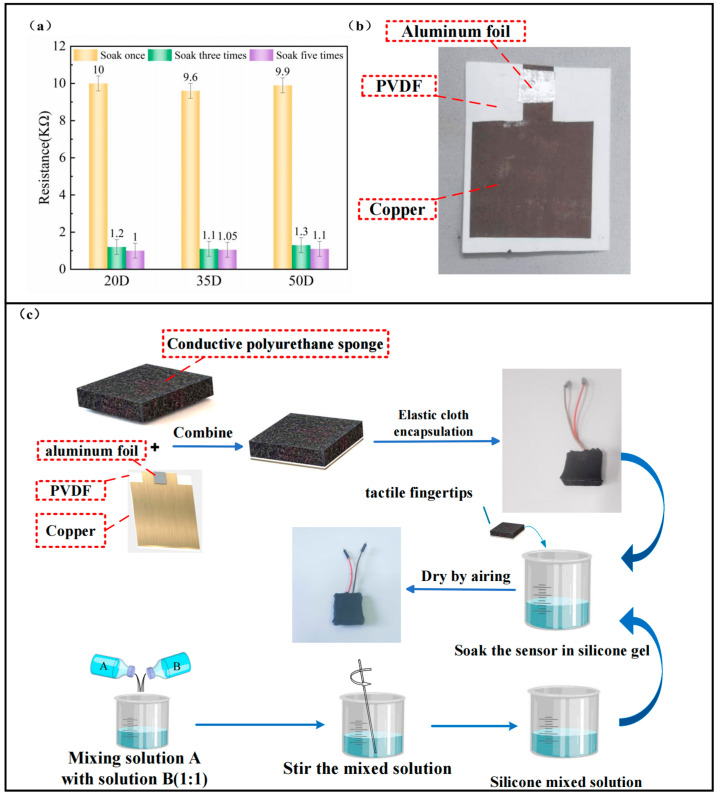
(**a**) Comparison of initial resistance of RGO-PUFs with different number of soaks; (**b**) schematic diagram of a PVDF film after deposition of a Cu film, wherein the aluminum foil serves to hold the Dupont wire in place; (**c**) haptic fingertip production flowchart.

Remarkably, we can flexibly adjust the electrical and mechanical properties of the RGO-PUFs based on changing the soaking time and the number of soakings. In addition, due to the simple and easy manufacturing process, we can manufacture RGO-PUFs of the desired size and properties in large quantities (see Figure S3, Supporting Information). Figure 3a shows the resistance of RGO-PUFs for different numbers of immersions. We can see that the RGO-PUFs with more time and a greater number of immersions have better electrical properties, but the mechanical properties are slightly degraded (deformation occurs). The resistance of the polyurethane sponge soaked once is around 10 kΩ. The resistance of polyurethane sponge soaked three times and five times can reach about 1 kΩ, and the resistance value is significantly improved.

### 2.3. Preparation of Sensors

In this paper, a PVDF membrane with a thickness of 100 μm is selected as the dielectric layer part of the haptic fingertip. On the one hand, the PVDF membrane is highly sensitive to physical quantities such as pressure and strain, and when paired with the graphene urethane sponge, it can lead to a dramatic increase in tactile fingertip sensitivity; on the other hand, PVDF membranes offer excellent corrosion and impact resistance, extending the life of tactile fingertips.

Magnetron sputtering produces high-quality, dense, uniform films with good adhesion and low defect rates. In this paper, magnetron sputtering is used to deposit the lower-electrode copper on the surface of PVDF film. The lower electrode is made to bond with the dielectric layer. This method produces an insulating layer that is better stabilized, lighter in weight, and takes up less space when applied to a haptic fingertip. In this paper, a magnetic sputtering device (SKY Technology Development Co., Ltd. Chinese Academy of Sciences, Beijing, China), JGP450A, was used to deposit thin copper films on PVDF films. Before sputtering, the PVDF membrane was ultrasonically cleaned using deionized water. The sputtering air pressure selected for this experiment was 2 pa, the sputtering power was 85 w, and the sputtering time was 1 h. Cu films were deposited on the PVDF film, as shown in Figure 3b. The thickness of the copper film produced by this method is 10 μm. The resistivity can reach 10 uΩ/cm.

We used an impregnated RGO-PUF as the upper electrode to fabricate the haptic fingertip samples. Here, the size of the RGO-PUF was chosen as 20 mm × 20 mm × 5 mm. Remarkably, selecting any size of RGO-PUF will not affect the performance of the tactile fingertips, and we can vary the size of the RGO-PUF as needed. The PVDF film was cut into 20 mm × 20 mm squares via laser cutting, as the PVDF film formed after laser cutting is more precise and avoids physical deformation of the material. In addition, we encapsulated it with a well-insulated black Carlisle fabric that isolates the tactile fingertips from the external environment. Finally, we encapsulated the haptic fingertip by applying a thinner layer of silicon rubber to the outer layer. The final haptic fingertip produced measured 20 mm × 20 mm × 6 mm. The fabrication process is shown in Figure 3c. The haptic fingertip weighed only 0.77 g.

## 3. Results

### 3.1. Pressure-Sensing Properties of the Sensor

**Figure 4:**
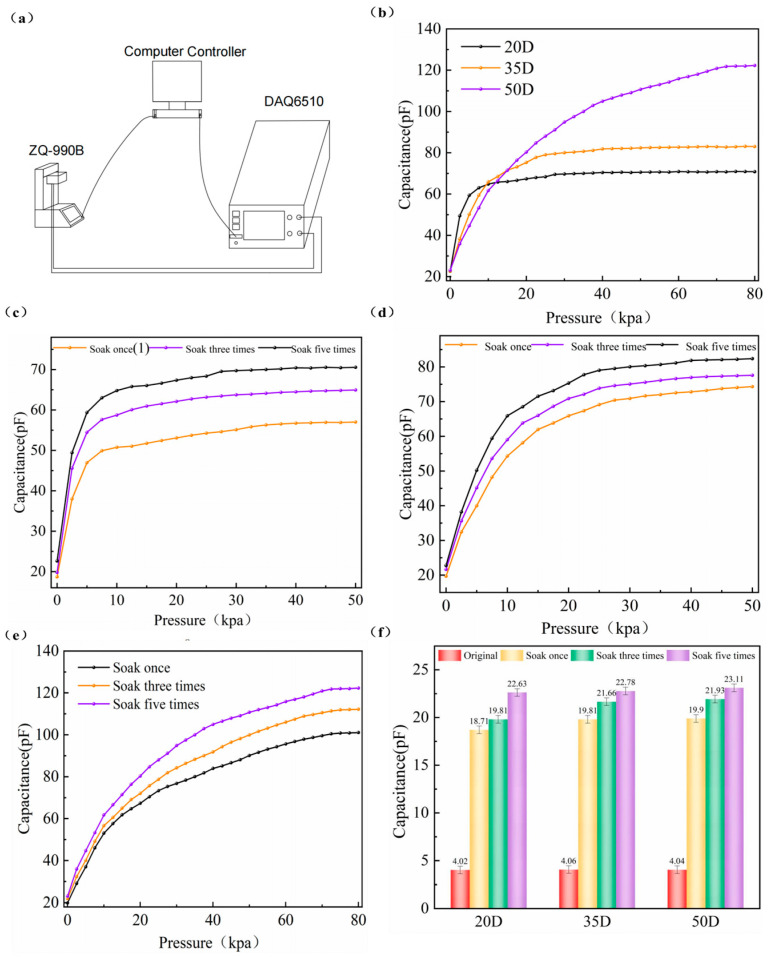
Schematic diagram of the sensing performance test pictures and experimental setup for tactile fingertips: (**a**) schematic diagram of the experimental setup; (**b**) the C–P curves of haptic fingertips made of RGO-PUFs of different hardnesses under five times of soaks and different applied pressures; (**c**) c–p curves of haptic fingertip samples (RGO-PUF hardness: 20D) at different numbers of soaks; (**d**) c–p curves of haptic fingertip samples (RGO-PUF hardness: 35D) at different numbers of soaks; (**e**) c–p curves of haptic fingertip samples (RGO-PUF hardness: 50D) at different numbers of soaks; (**f**) baseline capacitance maps of different tactile fingertip samples.

After the conclusions drawn from the above simulations, we equipped a pressure-capacitance test system to conduct pressure tests on the haptic fingertips. The test system consists of a universal testing machine ZQ-990B (purchased from Dongguan Wisdom Precision Instrument, Dongguan, China), a multimeter DAQ6510 (purchased from Keithley, Shanghai, China) to measure the change of capacitance value, and a computer control. It is shown in Figure 4a below. Keithley multimeter is characterized by large range, high accuracy, and multiple channels. With the multimeter testing software, it can convert, read, and store the capacitance signal realistically. We used the RGO-PUF with different hardnesses soaked out before to make 20 mm × 20 mm × 6 mm tactile fingertip samples to test its performance.

Sensitivity is the degree of sensitivity of a sensor to changes in the input signal. It indicates the smallest amount of change that the sensor can detect and measure. The sensor sensitivity is calculated as

(1) $$S=\frac{\Delta p}{\Delta c}=\frac{c_{1}-c_{0}}{c_{0}p},$$

where P denotes the applied pressure, and C1 and C0 denote the capacitances with and without applied pressure, respectively.

Figure 4b exhibits the pressure–capacitance variation of graphene urethane sponges with different hardnesses. We can see that there is a significant elevation in capacitance values in the low-pressure range for haptic fingertips made from graphene urethane sponges of different hardnesses. This highlights the highly sensitive properties of the tactile fingertip at low pressures. Haptic fingertips made of graphene urethane sponge with a hardness of 20 D have more-pronounced capacitance changes when subjected to low pressure. It can detect much smaller forces. The slope of the curve is the largest in 0~12.5 kpa; this stage is mainly manifested if the flexible tactile fingertip is pressurized by the two poles of the plate spacing. In this case, the sensitivity of the tactile fingertip was up to 1.089 kpa^−1^ in the load range of 0 to 0.25 kpa. Sponges with a hardness of 50D have a wider measuring range of up to 75 kpa. The 20D- and 35D-hardness sponges have stabilized their capacitance values at 40 kpa pressure. Figure 4c–e further investigate the pressure response curves of graphene urethane sponges—which were fabricated into tactile fingertips—at different numbers of soaks. The sensitivity of the haptic fingertip using an RGO-PUF of 20D hardness was 180 times higher than that using an insulating polyurethane sponge (conventional capacitive sensors using an insulating polyurethane sponge as a dielectric layer). The high-hardness RGO-PUF is subjected to pressure with relatively little deformation between the two polar plates. It can measure a wider pressure range, but the change in spacing between the two polar plates is not significant, and the sensitivity decreases slightly at low pressures compared to sponges of lower hardness. RGO-PUFs that are soaked more often have higher sensitivity over their pressure range, but the range does not change. We can use different hardnesses of graphene urethane sponges depending on the application scenario. In addition, we performed a linear fit for the RGO-PUF haptic fingertip samples immersed five times (see Figure S6, Supporting Information). Figure 4f plots the baseline capacitance of RGO-PUF at different hardnesses, and we can see that as the number of soaks rises. The baseline capacitance of the graphene urethane sponge increases significantly, up to a factor of 5–6, which makes the haptic fingertip robust to parasitic capacitance in the readout circuitry; therefore, it has a better signal-to-noise ratio.

**Figure 5:**
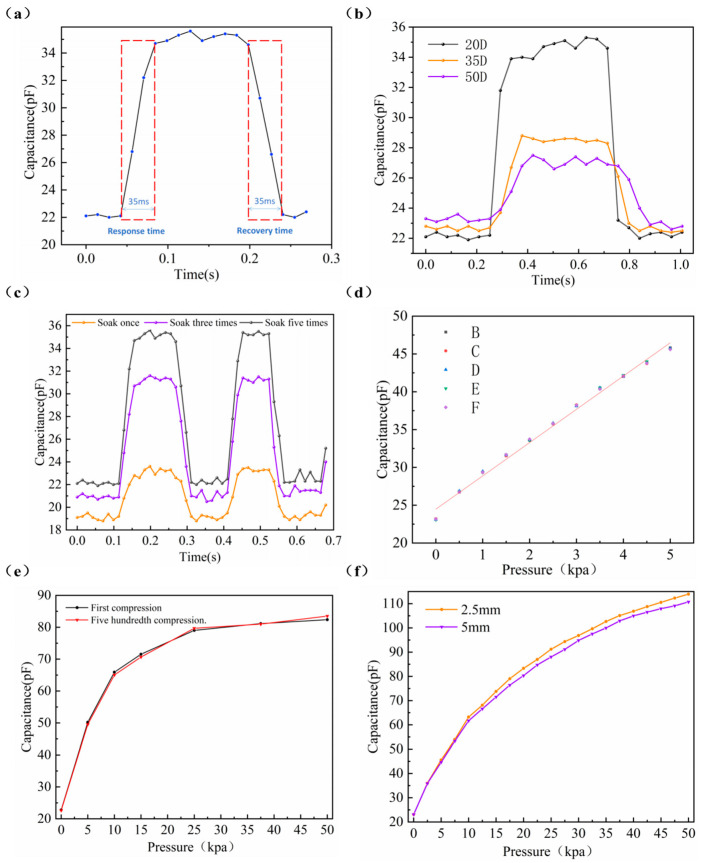
Sensing performance test pictures for haptic fingertips: (**a**) dynamic response and recovery characterization plots of tactile fingertip samples; (**b**) comparison of dynamic response and recovery characteristics of haptic fingertips made of graphene urethane sponge with different hardnesses; (**c**) comparison of dynamic response and recovery characteristics of haptic fingertips made of RGO-PUF with different numbers of soaks; (**d**) the C–P curves of tactile fingertip samples measured at five consecutive presses; (**e**) repeatability testing of tactile fingertip samples under different applied pressures; (**f**) the C–P curves of tactile fingertip samples of different thicknesses at different pressures.

Dynamic response time reflects the ability of a flexible sensor to respond to an applied load. This experiment used a pressure of 2.5 kpa and a loading rate of 10 mm/s to press the tactile fingertips. Figure 5a demonstrates the response time and recovery time of the 20D hardness sponge under five soaks, and its dynamic response time and recovery time can reach 35 ms. We compared the dynamic response of RGO-PUFs with different hardnesses after five immersions, as shown in Figure 5b. It can be seen that the 20D hardness sponge has the fastest dynamic response time and recovery time, the 35D hardness sponge is second, and the 50D hardness sponge has the longest dynamic response time. At the same time, we compared the dynamic response times of RGO-PUFs of the same hardness for different numbers of soaks, as shown in Figure 5c. As analyzed above, tactile fingertips have a fast dynamic response speed. The number of soakings of the RGO-PUF did not significantly correlate with the dynamic response time of the haptic fingertip. The lower-hardness graphene urethane sponge is made into a haptic fingertip with a faster dynamic response time.

Repeatability characteristics reflect the longevity and durability of a flexible sensor. The experiment used a loading rate of 10 mm/s and an application of 2.5 kpa to press the tactile fingertips. We chose an RGO-PUF that was soaked five times and has a hardness of 35D. Figure 5d shows the pressure response of the tactile fingertip sample over five consecutive measurements. The pressure response of five measurements in the range of 0 ~ 5 kpa also shows excellent agreement. To further measure the repeatability of the haptic fingertip, we performed 500 presses on the haptic fingertip using a press. The output performance curve did not undergo significant deformation, as shown in Figure 5e. The repeatability of the sponge did not change significantly after the sponges underwent redox. We again compared the repetition properties of RGO-PUFs of different hardnesses (see Figure S5, Supporting Information). As can be seen, there is no significant difference in the repetition characteristics, demonstrating the good recovery properties of the haptic fingertip, which is able to produce stable, continuous, and highly repetitive signals at a variety of low-frequency signals. In addition, we tested two different thicknesses of haptic fingertip samples (RGO-PUF with thicknesses of 2.5 mm and 5 mm and a hardness of 50D were used, respectively, and soaked five times), as shown in Figure 5f. As can be seen, the thickness of the two tactile fingertip samples differed by a factor of two, but the difference in sensitivity was not significant. The tactile fingertip samples with small thicknesses were slightly more sensitive. The reason for this is that they have similar workspaces (see Figure 1d); therefore, their sensitivity differences are small. We can make tactile fingertips of different thicknesses with similar sensitivity for different applications.

### 3.2. Application Experiments

In order to further verify the excellent performance of the haptic tactile fingertip in low-pressure detection, we mounted the tactile fingertip on a mycobot pro 600 (Elephant Robotics Technology Co., Ltd., Shenzhen, China.) robotic gripper and performed object grasping experiments. In order to highlight the performance of the haptic fingertip more, we chose jelly (weight 20 g) and live silkworm (weight 2 g) as grasping objects. In this experiment, because the graphene urethane sponge with a hardness of 20D has the highest sensitivity in gripping low-weight objects, we used a graphene urethane sponge soaked five times and with a hardness of 20D as the upper electrode of the tactile fingertip.

**Figure 6:**
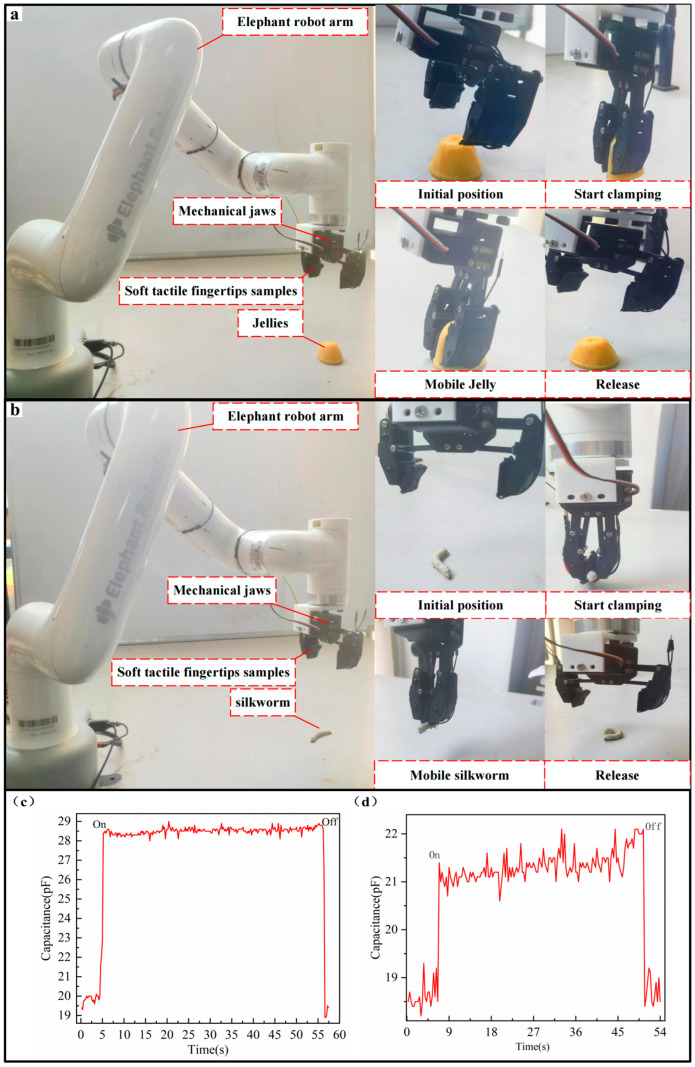
(**a**) Schematic diagram of the robot mycobot 600 for clamping jellies; (**b**) schematic diagram of the use of the robot mycobot 600 for clamping silkworms; (**c**) changes in capacitance of haptic fingertip samples when holding jelly; (**d**) changes in capacitance of haptic fingertip samples during clamping of live silkworms.

We made two of the haptic fingertips according to the shape of the robot’s claw, fixed them on the robot, and measured the capacitance value of the haptic fingertips when the gripped object was firm and undamaged using a Keithley multimeter DAQ6510. After that, we wrote a grasping programme and performed automatic robot grasping and releasing experiments. The object is clamped and moved to another position. It is worth pointing out that the shape of the haptic fingertip is unrestricted, and we can change the shape of the haptic fingertip by changing the shape and size of the RGO-PUF. The schematic diagram of the clamping jelly experiment is shown in Figure 6a. The capacitance change of the haptic fingertip can be clearly seen during the clamping of jelly, as shown in Figure 6c. We can see that the haptic fingertip can reliably detect very small masses and has excellent dynamic response with high sensitivity. Moreover, the jelly did not deform before and after clamping by the robotic claw, demonstrating that the elastic porous electrodes have good energy-absorption and shock-absorption capabilities. In addition, the experimental procedure for grasping live silkworms was the same as that for clamping jelly. The schematic diagram of the gripping silkworms experiment is shown in Figure 6b. In the experiment, the capacitance value of the haptic fingertip also changed significantly, and the live silkworm was not damaged. The capacitance change is shown in Figure 6d, which shows that the new haptic fingertip still has a significant capacitance change when detecting very low pressure (0.02 N). The experimental results show that the haptic fingertip has excellent performance in grasping ultra-light and ultra-low-modulus objects, similar to that of human fingers, and can perform dynamic grasping without damaging the objects.

## 4. Conclusions and Discussion

In conclusion, we have designed an elastic porous material preparation method with adjustable conductivity, pore density, and elastic modulus, and based on this, we have designed a soft tactile fingertip for robotics that is gentle, highly sensitive, and has adjustable range. By means of the redox method, we succeeded in applying graphene on the skeleton surface of polyurethane sponge, avoiding the problem of performance degradation of the haptic fingertips due to solution immersion. This tactile fingertip has high sensitivity (~1.089 kpa^−1^), extremely fast dynamic response (~35 ms), a very low detection limit (0.02 N), excellent durability over 500 cycles, 5–6 times increased baseline capacitance, and good shock absorption. The haptic fingertip samples were mounted on the rigid manipulator of the mycobot pro 600 robot for grasping experiments. The experimental results show that the tactile fingertips have excellent performance—similar to that of a human finger—when grasping ultra-light and ultra-low-modulus objects.

## Data Availability

Data will be made available on request.

## Supplementary Materials

The following supporting information can be downloaded at https://www.mdpi.com/article/10.3390/mi15050628/s1: Figure S1: Photographs of polyurethane sponge before and after immersion in graphene oxide and HI solutions; Figure S2: Elemental diagram of RGO-PUF with 50D hardness after 5 soakings; Figure S3: Morphological changes of RGO-PUFs with different hardnesses under different number of soaks; Figure S4: Schematic diagram of the experimental setup. The whole system consists of ZQ-990B, DAQ6510, and a computer. ZQ-990B is connected to the computer through wires, and the computer sets the parameters to regulate the pressure; DAQ6510 is connected to the computer through the USB cable, and the collected capacitance value is fed back to the computer; Figure S5: Linear fit plots of fingertip tactile samples of different hardness of RGO-PUF under five immersions. (a) RGO-PUF with 20D hardness. (b) RGO-PUF with 35D hardness. (b) RGO-PUF with 50D hardness. Figure S6: Repeatability testing of tactile fingertip samples under different applied pressures: (a) repeatability of RGO-PUF with 20D hardness after five soaks; (b) repeatability of RGO-PUF with 50D hardness after five soaks.
