# Supplementary material for: A Soft Robot Tactile Finger Using Oxidation-Reduction Graphene–Polyurethane Conductive Sponge
Source: Micromachines (Basel). 2024 May 7;15(5):628. doi: 10.3390/mi15050628 (PMC11123064; doi:10.3390/mi15050628)
Supplement: Supplementary file 1 [file micromachines-15-00628-s001.zip › micromachines-2980608-supplementary.pdf]

micromachines

Article

A soft robot tactile finger using qxidation-reduction graphene-polyurethane conductive sponge

Hangze Li [1], Chaolin Ma [1], Jinmiao Chen [1], Haojie Wang [1], Xiao Chen [1], Zhijing Li [2] and Youzhi Zhang [1,*]

1 School of Mechanical and Electrical Engineering, Wenzhou University, Wenzhou, 325025, China; lihangze2023@163.com(H.L.);mchaolin2023@163.com(C.M.);chenjinmiao22@163.com(J.C.)wanghaojie020@163.com(H.W.);lscx09@163.com(X.C.)
2 School of Information and Electrical Engineering, Hunan University of Science and Technology,Xiangtan, 411201, China,lizhijingwei@163.com;
* Correspondence: zhangyouzhi@wzu.edu.cn

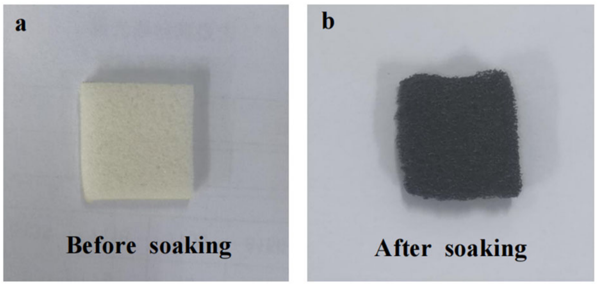

Figure S1. Photographs of polyurethane sponge before and after immersion in graphene oxide and HI solutions.

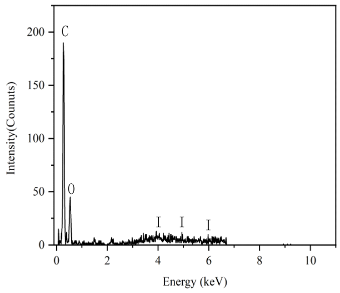

Figure S2. Elemental diagram of RGO-PUF with 50D hardness after 5 soakings.

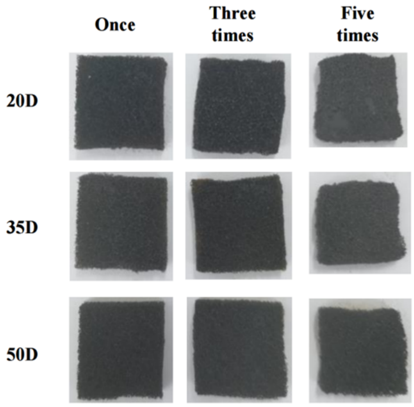

Figure S3. Morphological changes of RGO-PUF with different hardnesses under different number of soaks.

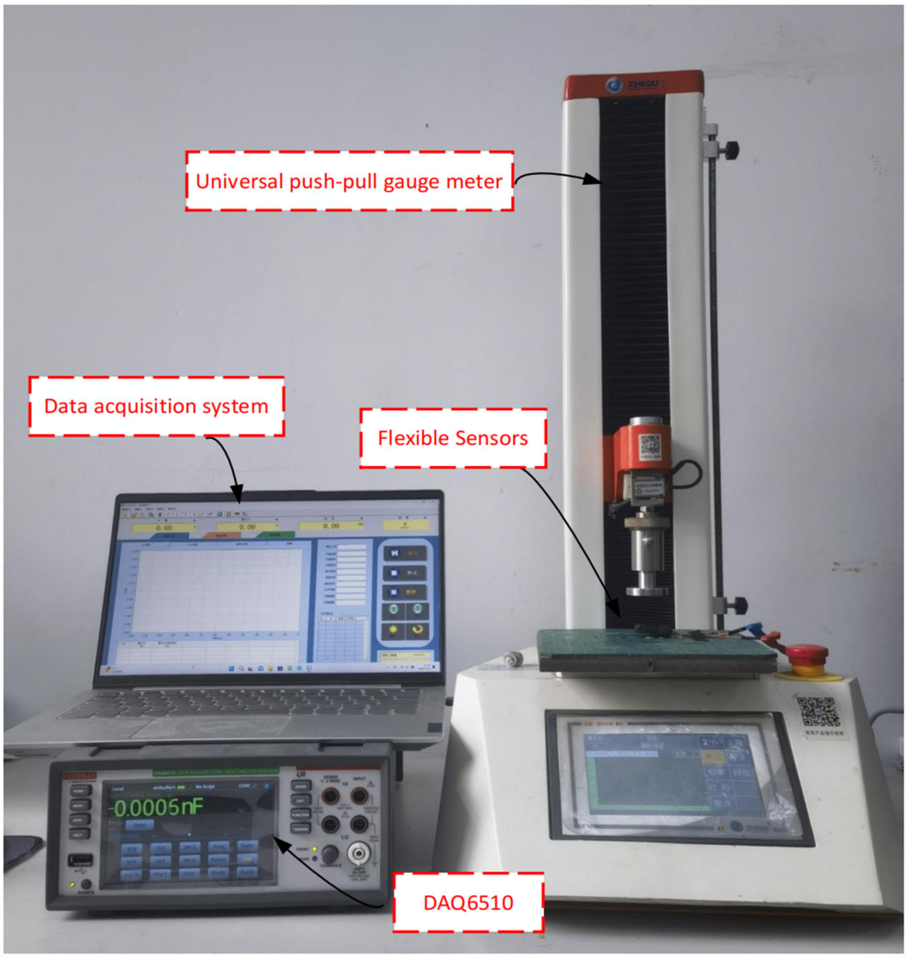

Figure S4. Schematic diagram of the experimental setup.The whole system consists of ZQ-990B, DAQ6510 and computer. ZQ-990B is connected to the computer through wires, and the computer sets the parameters to regulate the pressure; DAQ6510 is connected to the computer through the USB cable, and the collected capacitance value is fed back to the computer.

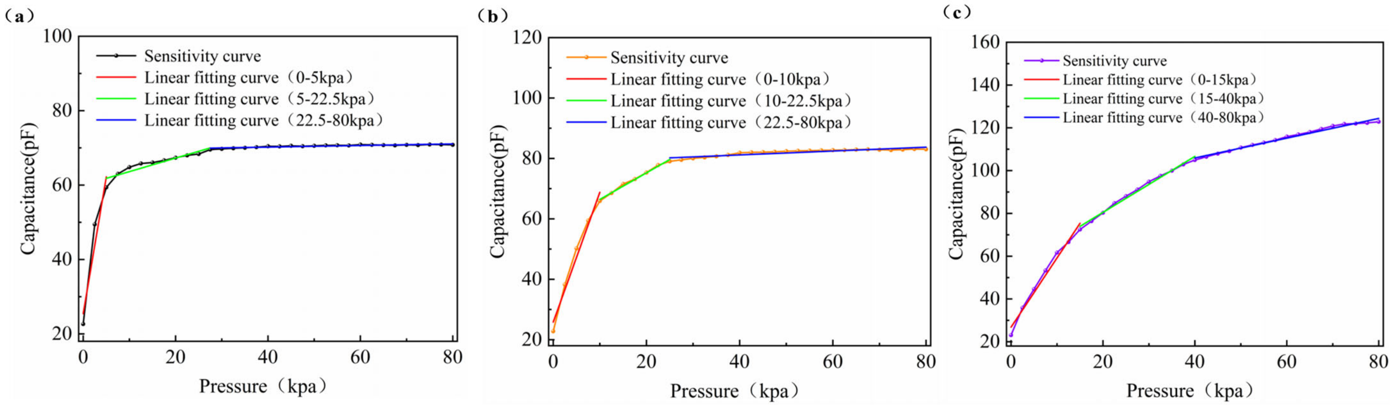

Figure S5. Linear fit plots of fingertip tactile samples of different hardness of RGO-PUF under five immersions. (a) RGO-PUF with 20D hardness. (b) RGO-PUF with 35D hardness. (b) RGO-PUF with 50D hardness.

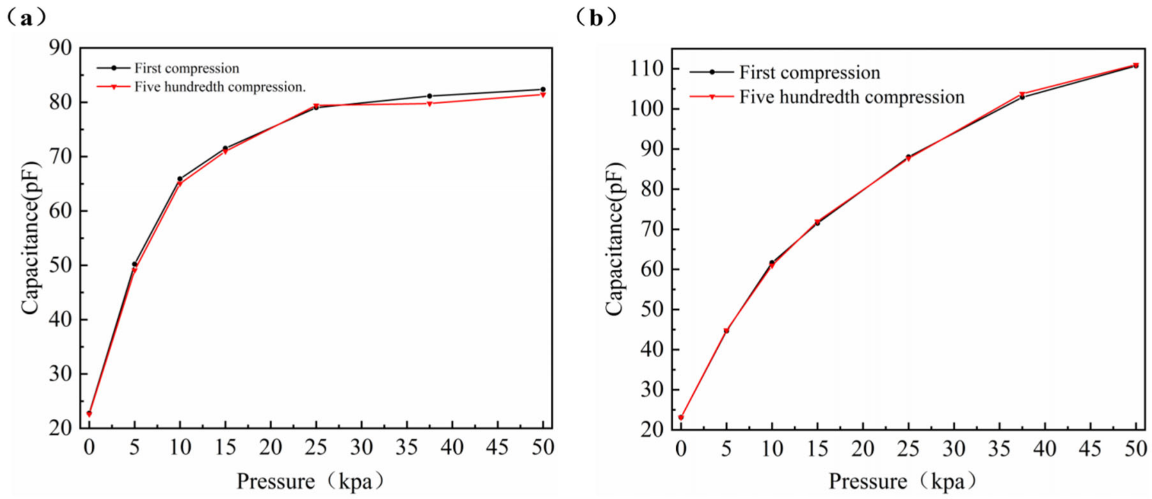

Figure S6. Repeatability testing of tactile fingertip samples under different applied pressures. (a) Repeatability of RGO-PUF with 20D hardness after 5 soaks. (b) Repeatability of RGO-PUF with 50D hardness after 5 soaks.
